# Ectopic Cushing Syndrome in Adenocarcinoma of the Lung: Case Report and Literature Review

**DOI:** 10.7759/cureus.14733

**Published:** 2021-04-28

**Authors:** Rana Al-Zakhari, Safa Aljammali, Basma Ataallah, Svetoslav Bardarov, Philip Otterbeck

**Affiliations:** 1 Internal Medicine, Richmond University Medical Center, Staten Island, USA; 2 Internal Medicine, Zucker School of Medicine at Mather, Port Jefferson, USA; 3 Internal Medicine, Northwell Health Mather Hospital, Port Jefferson, USA; 4 Pathology and Laboratory Medicine, Richmond University Medical Center, Staten Island, USA

**Keywords:** paraneoplastic syndromes, ectopic cushing’s syndrome, adenocarcinoma

## Abstract

Paraneoplastic syndromes are rare disorders that occur with many types of tumors. Ectopic cushing syndrome (ECS) is the second most common paraneoplastic syndrome that is only seen in 1-5% of all small cell lung cancers (SCLC), with limited papers reporting this syndrome since it was first described by Brown in 1928 or in carcinoid tumors. It is also found to be associated to a lesser extent with pheochromocytoma, thymic tumors, pancreatic carcinoma, and anaplastic thyroid carcinoma. While lung adenocarcinoma is the most common histological type of lung neoplasms, it is seldom associated with Cushing syndrome. In this article, we describe a patient who initially presented with Cushing syndrome and found to have adenocarcinoma of the lung.

## Introduction

Adenocarcinoma of the lung is the most common primary non-small cell lung carcinoma in the United States. Despite the decline in its incidence and mortality, it remains the leading cause of cancer death in the United States [1]. Adenocarcinoma is rarely observed before the age of 20, and the median age of its diagnosis is approximately 71 years. Over the past two decades, this particular cancer has replaced squamous cell carcinoma as the most common non-small cell carcinoma of the lung [2]. Paraneoplastic syndromes can be associated with benign or malignant neoplasms. They are commonly seen in association with different types of malignancies such as neuroendocrine carcinomas and rarely adenocarcinomas [3]. When present, such paraneoplastic effects can be attributed directly to the neoplastic process and are expected to resolve as the neoplasms resolve during chemotherapy [4]. Thus, they can be used as a follow-up marker and screening for recurrence.

## Case presentation

A 60-year-old male patient with a history of post-traumatic stress disorder, hypertension, and type 2 diabetes mellitus was brought by Emergency Medical Services to the emergency department. Upon arrival, he reported that he fell, and was unable to get up. He also admitted to alcohol use disorder and a 30-year smoking history.

**Figure 1:**
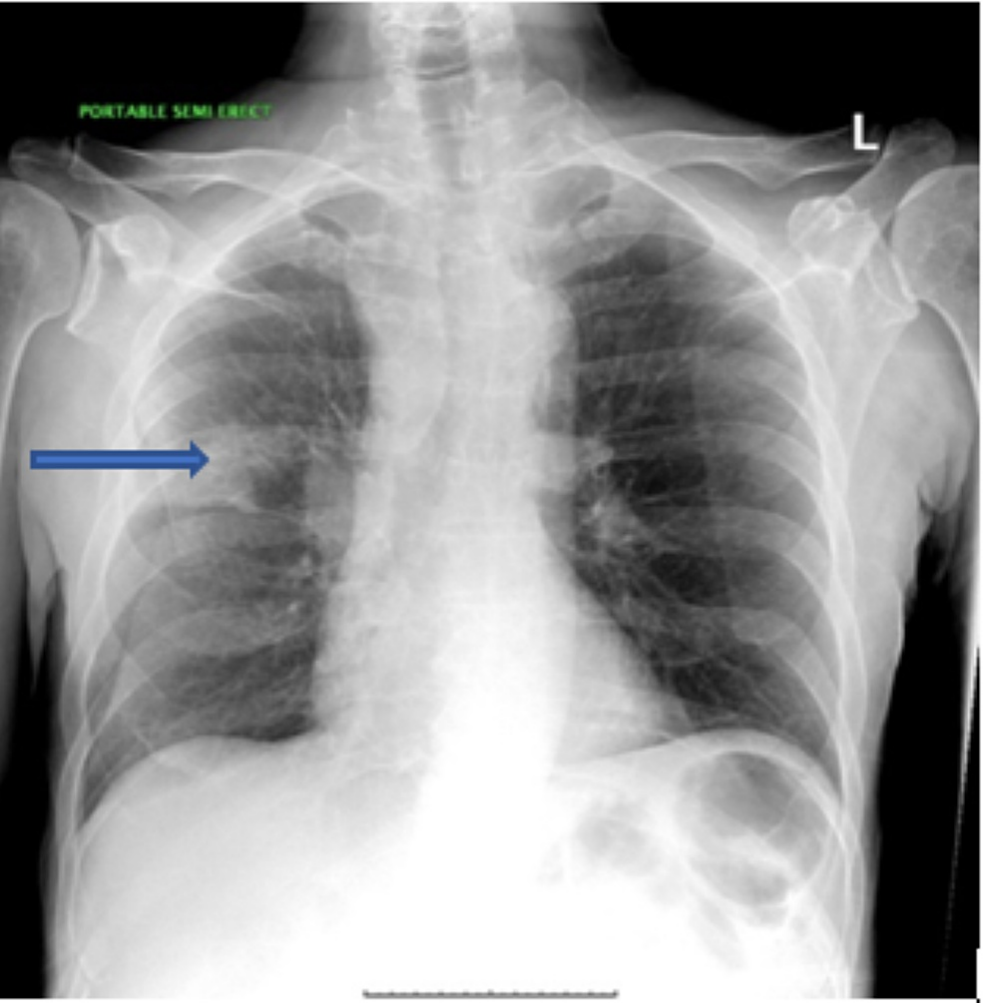
Chest radiograph demonstrated a 3 cm right upper lobe mass above the minor fissure (blue arrow) with possible infiltrative process extending toward the right hilum and prominent right hilum and right paratracheal soft tissues.

The patient reported watery diarrhea for approximately three to four days duration. He also reported dry cough for an unknown duration. However, he denied shortness of breath, fever, abdominal pain, vomiting, chest pain, or urinary symptoms. His physical examination revealed cachexia and a disheveled appearance. In the emergency department, computed tomography (CT) of the head showed no acute intracranial findings. The chest radiograph showed a three-centimeter right upper lobe mass over the minor fissure (Figure 1).

Biochemistry assessment revealed severe hypokalemia (2.0 mmol/L, ref 3.5-5.0 mmol/ L), hypercarbia (54 mmol/L, ref 21-32 mmol/L), hypocalcemia (7.4 mg/dL, ref 8.4-10.2 mg/dL), hypochloremia (83 mmol/L, ref 98-107 mmol/L), metabolic alkalosis with venous bicarbonate of 48 mmol/dl and arterial blood pH of 7.58, hyperglycemia (random blood-sugar range of 150 to 250 mg/dl), early morning cortisol (138.1 ug/dl with reference range 5.27 - 22.45 ug/dl), serum adrenocorticotropic hormone (ACTH) was 714 pg/mL (ref 6 - 50 pg/mL). Follow-up 24h-urine-free cortisol was 7856.5 mcg/24h (reference range 4.0 - 50.0 mcg/24h). Moreover, 1 mg overnight dexamethasone suppression result was grossly positive with a morning cortisol of more than 120.0 ug/dl (reference range: 5.27 - 22.45) establishing the diagnosis of Cushing syndrome.

Electrocardiogram (EKG) revealed an extended QTc of 579 msec. He was simultaneously diagnosed with aspiration pneumonia and treated with intravenous antibiotics. At this point, aggressive repletion of potassium was initiated.

**Figure 2:**
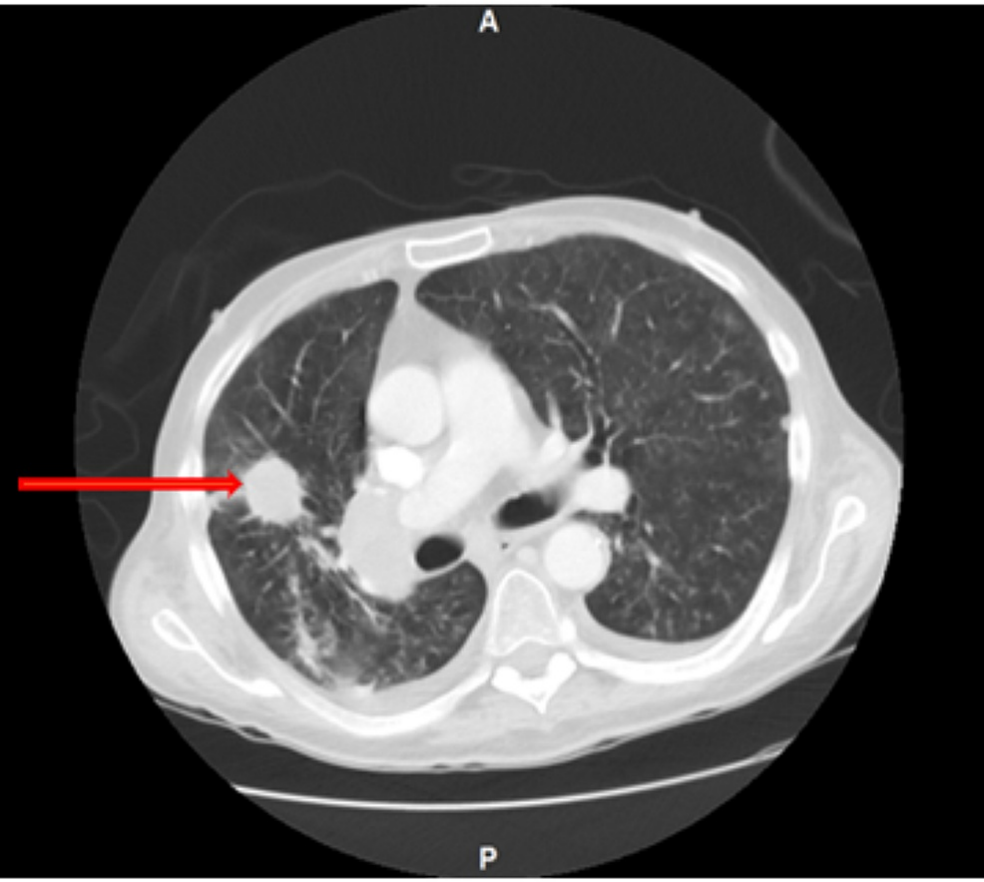
Computed tomography (CT) of the chest without contrast shows a speculated 2.7 cm lesion in the right upper lobe (red arrow) with right hilar and paratracheal lymphadenopathy measuring 3.8 cm and 4.7 x 3.7 cm, respectively.

Magnetic resonance imaging (MRI) of the head was performed, and it did not show any hypothalamic or pituitary lesions. Computed tomography (CT) of the chest without contrast additionally evaluated the pulmonary findings on chest X-ray and a spiculated 2.7 cm nodule was found at the right upper lobe, suspicious for malignancy with an adjacent 1.4 cm lesion (Figure 2).

Computer tomography (CT) of pelvis and abdomen with contrast was also done and demonstrated a fungating endoluminal mass at the sigmoid colon. Colon biopsy derived from rectal, sigmoid, descending, and ascending polyps, demonstrated superficial pieces of villous adenoma having focal high-grade dysplasia. Other findings were unremarkable. 

A CT-guided core biopsy of the right upper lobe lung mass was performed and demonstrated a neoplastic process composed of large epithelioid cells with prominent nucleoli arranged in solid and acinar areas.

**Figure 3:**
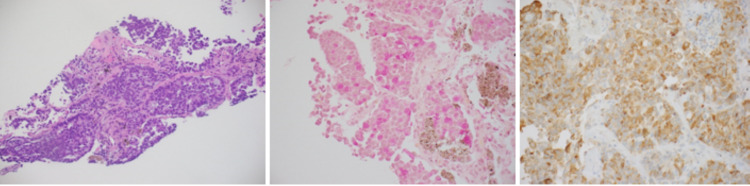
Histopathology of the right upper lobe lung mass was performed and showed pulmonary adenocarcinoma, moderately to poorly differentiated (left slide). The tumor cells were positive for CK7, TTF-1, and Napsin A (weak and focal), and negative for CK20 or p40 (middle slide). Mucin stain was positive (right slide).

The tumor cells were positive for CK7, TTF-1, with weak Napsin A stain, while negative for CK20 or p40. Mucin stain was positive. These studies support the diagnosis of pulmonary adenocarcinoma (Figure 3).

Additional immunostaining for ACTH demonstrated a strong cytoplasmic stain in the neoplastic cells.

Based on the clinical, radiologic, and pathologic findings, the patient was diagnosed with poorly differentiated adenocarcinoma of the lung with ectopic Cushing syndrome, the patient was started on ketoconazole 200 mg three times a day. Five days later, early morning cortisol level reduced significantly to 75.0 ug/dl. The patient was to be seen by hematology/oncology as an outpatient to initiate immunotherapy/chemotherapy.

## Discussion

Ectopic Cushing syndrome (ECS) is defined by the production of ACTH by a tumor originating outside the pituitary glands. This entity was first described in 1965 and it accounts for 5%-10% of cases of spontaneous Cushing's syndrome [5]. Most of these tumors have been small-cell tumors of the lung or carcinoid tumors, and less frequently, neoplasms emerging from the embryonic structures and tissues with endocrine function, including pheochromocytoma, thymic tumors, pancreatic carcinoma, and anaplastic thyroid carcinoma [6]. It has been reported in the literature that cases of ectopic adrenocorticotropic hormone production have been associated with the adenocarcinomas of the gallbladder, salivary glands, colon, and possibly prostate [7-10]. However, only a few instances of this condition have been linked to adenocarcinoma of the lung [6,11].

The common cushingoid features may not be present in cases of ECS. However, cushingoid features are often present with carcinoid tumors and generally present with electrolyte disturbances in cases other than carcinoid tumors due to their sudden onset and aggressive nature [12]. As our patient presented, hypokalemia and metabolic alkalosis are the usual findings that suggest the presence of increased ACTH production and lead to subsequent confirmation testing. [5]

Patients suffering from ectopic adrenocorticotropic hormone production have high ACTH levels (>20 ng/L), high cortisol levels that are not suppressed by high dexamethasone dosages (8 mg per day), and they have negative response of the pituitary gland to the corticotrophin-releasing hormone. In a few examples, patients having excess ACTH production can be appropriately suppressed by high-dose dexamethasone. In those instances, the inferior petrosal sinus sampling test might be an effective way to establish the diagnosis [13].

When the diagnosis of an ectopic ACTH-secreting tumor is established, the cornerstone of management is to treat the underlying neoplasm. But medical interventions with glucocorticoid synthesis inhibitors, including octreotide, metyrapone, mitotane, and ketoconazole are optional. Ketoconazole blocks corticosteroid production by restraining 17-hydroxylase and 11-hydroxylase. It remains the primary treatment approach due to drug availability and favorable side effect profile. Spironolactone and potassium replacement could be used as well [14,15].

It has been reported in the literature that patients with ECS have increased mortality due to the high levels of cortisol and marked suppression of the immune system. This can make patients more prone to septicemia and opportunistic infections. Some researchers suggest that lowering the cortisol levels before implementing curative treatments including chemotherapy or surgery might decrease morbidity and mortality [6].

## Conclusions

Sudden onset of signs and symptoms consistent with Cushing syndrome, in a previously healthy patients should have a high suspicion index for an ectopic ACTH production. Though ectopic adrenocorticotropic hormone syndrome with adenocarcinoma of the lung is rare, it is important to consider it within the differential diagnosis of any adenocarcinoma of the lung presenting electrolytes disturbance, hypertension, and hyperglycemia. The available data in this area are quite limited, so additional work should be done in this area to establish the true prevalence of this problem. It might also be helpful to evaluate the impact of ACTH as a marker to assess the response to the cancer treatments in similar cases or as a follow up marker of recurrent malignancy or metastasis.
